# Proteins à la carte: riboproteogenomic exploration of bacterial N-terminal proteoform expression

**DOI:** 10.1128/mbio.00333-24

**Published:** 2024-03-21

**Authors:** Igor Fijalkowski, Valdes Snauwaert, Petra Van Damme

**Affiliations:** 1 iRIP Unit, Laboratory of Microbiology, Department of Biochemistry and Microbiology, Ghent University, Ghent, Belgium; University of Würzburg, Würzburg, Germany; Wadsworth Center, Albany, New York, USA

**Keywords:** alternative translation initiation, N-terminal proteoforms, N-terminal proteomics, riboproteogenomics, (retapamulin-assisted) ribosome profiling (Ribo-RET)

## Abstract

In recent years, it has become evident that the true complexity of bacterial proteomes remains underestimated. Gene annotation tools are known to propagate biases and overlook certain classes of truly expressed proteins, particularly proteoforms—protein isoforms arising from a single gene. Recent (re-)annotation efforts heavily rely on ribosome profiling by providing a direct readout of translation to fully describe bacterial proteomes. In this study, we employ a robust riboproteogenomic pipeline to conduct a systematic census of expressed N-terminal proteoform pairs, representing two isoforms encoded by a single gene raised by annotated and alternative translation initiation, in *Salmonella*. Intriguingly, conditional-dependent changes in relative utilization of annotated and alternative translation initiation sites (TIS) were observed in several cases. This suggests that TIS selection is subject to regulatory control, adding yet another layer of complexity to our understanding of bacterial proteomes.

**IMPORTANCE:**

With the emerging theme of genes within genes comprising the existence of alternative open reading frames (ORFs) generated by translation initiation at in-frame start codons, mechanisms that control the relative utilization of annotated and alternative TIS need to be unraveled and our molecular understanding of resulting proteoforms broadened. Utilizing complementary ribosome profiling strategies to map ORF boundaries, we uncovered dual-encoding ORFs generated by in-frame TIS usage in *Salmonella*. Besides demonstrating that alternative TIS usage may generate proteoforms with different characteristics, such as differential localization and specialized function, quantitative aspects of conditional retapamulin-assisted ribosome profiling (Ribo-RET) translation initiation maps offer unprecedented insights into the relative utilization of annotated and alternative TIS, enabling the exploration of gene regulatory mechanisms that control TIS usage and, consequently, the translation of N-terminal proteoform pairs.

## INTRODUCTION

In recent years, the rapid accumulation of novel genome sequencing efforts in bacteria has rendered manual genome annotation unfeasible. Despite continuous improvements, automatic genome annotation pipelines have repeatedly been shown to vastly underestimate the true complexity of bacterial genomes (1, 2). Annotation census efforts have revealed that among over 100 annotated prokaryotic genomes, up to 60% of genes suffered from incorrectly assigned translation initiation sites (TISs) with the longest possible open reading frame (ORF) often being preferentially selected *in silico* (3). This fact highlights the particular difficulty in accurately and comprehensively assigning TISs. Annotation pipelines have also demonstrated limited capacity in discovering novel protein families, as clearly evident for notoriously under annotated small ORFs (sORFs) (4–6), posing a risk of propagating errors persisting in current annotations (1, 7). Moreover, the currently employed tools exhibit relatively poor (~70%) agreement between predictions, and not all newly submitted genomes utilize the same state-of-the-art annotation tools (8). To add to this, the accuracy of prediction algorithms can be highly dependent on the properties of individual genomes, such as their guanine-cytosine (GC) content, impacting not only general gene detection but also TIS assignment. These inaccuracies further lead to erroneous predictions that do not accurately represent actual protein-coding genes or regions (9). Importantly, in the case of bacterial genomes, multiple TIS annotations within a single gene or transcript are generally not considered. Altogether, we and others have demonstrated that all these factors contribute to the persistent underestimation of the true complexity of bacterial genomes and their encoding proteomes, even in case of well-studied organisms (10).

The shortcomings of currently applied genome annotation strategies have gradually been addressed by continuous, experimental data-driven reannotation efforts. Owed to the recent developments in genomics, ribosome profiling (Ribo-seq) has provided an unprecedented view on bacterial translational landscapes by sequencing of mRNA fragments encapsulated within actively translating ribosomes (11–13). This technology has not only demonstrated pervasive translation outside of annotated genes but has also allowed for the correction of existing annotation errors (7, 11–16). The most recent additions to the bacterial genomics toolkit, retapamulin-assisted ribosome profiling (Ribo-RET) and antimicrobial peptide oncocin (Onc112) ribosome profiling, have enabled an in-depth examination of bacterial translation initiation landscapes (17, 18). With the recent advent of translation initiation mapping, high-throughput investigation of alternative TIS selection became feasible in bacterial models, similar to lactimidomycin-based TIS profiling permitting analogous studies in eukaryotic models (11). Such a systematic effort allows us to fully appreciate the repertoire of molecular protein forms expressed, including proteoforms originating from alternative in-frame TIS selection (and not proteolysis)—here referred to as N-terminal (Nt-)proteoforms—which posed a particular challenge to formerly employed detection methods (15, 16). In the absence of reliable genomics data on bacterial translation initiation, the repertoire of technologies suitable for proteoform-targeted studies has been limited. On the protein level, alternative Nt-proteoforms often share the great majority of their identifiable peptides, especially since an alternative TIS is often contained within the 3’ part of the coding sequence (CDS), rendering its unambiguous proteomic identification challenging. Therefore, unique Nt-peptides are often the sole means of confident Nt-proteoform detection. To this end, Nt-proteomics methods, jointly referred to as N-terminomics, have been the most successful in elucidating the landscape of Nt-proteome variation in bacteria (16). Complicating the assignment of N-termini to bacterial TIS further is the typical absence of Nt-protein modifications at mature bacterial protein N-termini, which, in the case of eukaryotic N-termini, serve as proxies for translation initiation (e.g., N-terminal acetylation). Previously, a subset of 11 genes displaying evidence of annotated as well as alternative initiation has been found in *Escherichia coli* utilizing a potent N-terminal proteomics technique (COFRADIC) in conjunction with actinonin treatment, effectively leaving true N-termini marked with formyl groups (15, 16). Studies that aim at correcting genome and concomitantly TIS annotations are thus undisputedly of central importance for improving our understanding of bacterial systems biology (19–21). Utilizing complementary translation (initiation) and proteomics (e.g., N-terminomics) data, an approach referred to as riboproteogenomics, has already shown great promise in this regard (15, 16, 22). Yet, further improvements in proteomics validation of genomic findings still remain an important challenge (10, 23).

Albeit well-established in eukaryotic models (14, 24, 25), only scarce examples of alternative Nt-proteoform production in bacteria have been described to date (26). However, a recent landmark study, reporting on *E. coli* Ribo-RET data sets, discovered 42 genes displaying evidence of annotated as well as alternative translation initiation, thereby reporting on the translation of gene-specific Nt-proteoform pairs at the genome-wide level for the first time (17). Moreover, limited studied cases reporting on the properties of individual members of Nt-proteoform pairs have uncovered both structural and functional implications for such alternative proteoforms (27, 28). It is conceivable that alternative translation initiation in bacteria can bear similar functions to previously described mammalian and plant alternative initiation events, allowing for a rapid response to environmental cues, regulating subcellular localization and protein stability, and serving distinct functions within multiprotein complexes (10, 25, 29, 30). In *Salmonella*, it has been shown that the simultaneous expression of two SsaQ proteoforms is required for the effective formation of the type III secretion system (T3SS) utilized for effector delivery into host cells (31). Nt-proteoforms were further shown to engage in unique protein–protein interactions (PPIs) and in the formation of alternative protein complexes, as reviewed by Fijalkowska et al. (10). In cyanobacteria, it has been shown that CcmM Nt-proteoforms serve distinct functions in the maturation of the quaternary Rubisco complex. Specifically, the short proteoform of CcmM (short CcmM or CcmM^S^) serves as structural component of Rubisco supercomplex, while the annotated, longer CcmM proteoform (CcmM^L^) serves as an anchor of the complex in the carboxysome lumen (32). Additionally, the Nt-truncated *E. coli* proteoform CheA^S^ engages in both phosphorylating and dephosphorylating complexes of the chemotaxis pathway, while CheA^L^ acts exclusively as a kinase (33). Moreover, two alternative TISs in the *E. coli infB* gene were shown to give rise to translation initiation factor 2 α and β isoforms, respectively (34). As a final representative example, the Nt-proteoforms GALLS^S^ and GALLS^L^ of *Agrobacterium rhizogenes* were shown to encode two essential components of the T-DNA complex required for plant transformation (35). Despite these intriguing findings, only a few systematic efforts have been made to comprehensively catalogue the repertoire of expressed bacterial Nt-proteoforms and to study the regulatory mechanisms governing their potential conditional (e.g., growth-phase-specific) expression. Given the functional implications of alternative Nt-proteoform expression, their investigation is crucial to elucidate the intricacies of bacterial systems biology and, by extension, to fully grasp the pathogenicity mechanisms displayed by infectious bacteria.

In this work, we conducted a systemic riboproteogenomic quest to explore the genome of the intensively studied model bacterial pathogen *Salmonella enterica* serovar Typhimurium (*S. Typhimurium*) for paired Nt-proteoform expression. Similar to most bacterial species, and despite major recent advances made by us and others, our understanding of the true genome complexity of *S*. Typhimurium is still incomplete (4, 16, 22, 36–38). In *S. Typhimurium*, Nt-proteoform mapping revealed about 50 genes potentially encoding Nt-proteoform pairs, laying the foundation for their functional characterization and possible functional diversification. Specifically, Ribo-seq and Ribo-RET data generated across a panel of complementary growth conditions were repurposed to more comprehensively capture the range of expressed proteoforms—the full coding potential of the bacterial genome. By studying distinct bacterial growth phases and various environmental stresses, the value of using Ribo-RET data for studying regulation of Nt-proteoform expression was investigated, providing a first hint toward conditional proteoform expression alongside the potential diversifying biological functions of proteoforms. With the functional relevance of these understudied genomic elements, the study of so far uncharted translation (initiation) events opens a novel avenue into improving our understanding of bacterial systems.

## RESULTS

### Ribo-RET data for the detection of alternative initiation events leading to bacterial proteoform expression

**Fig 1:**
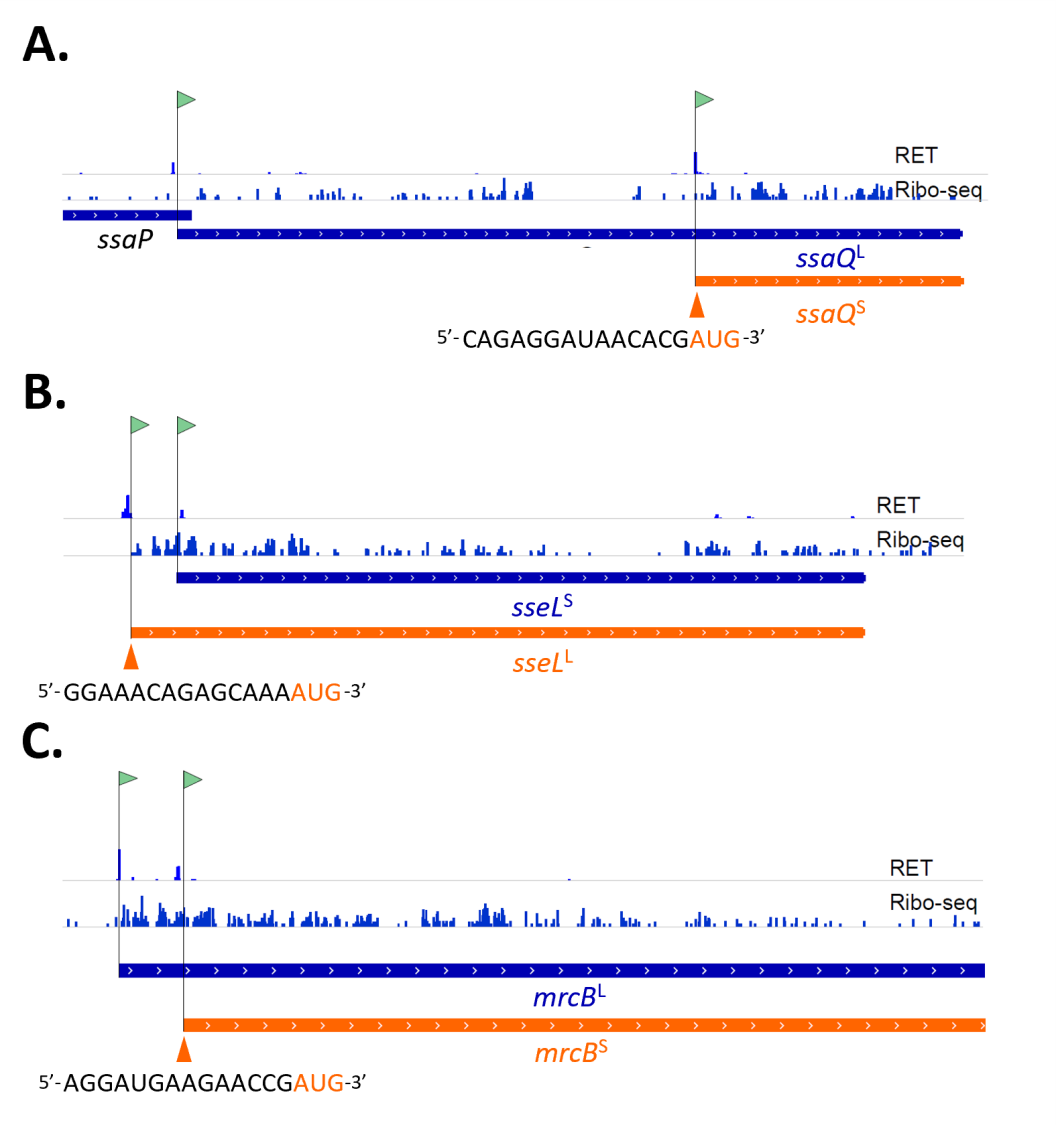
Ribo-seq unveils translation of Nt-proteoform pairs in *S.* Typhimurium. Ribo-RET (RET) and Ribo-seq signals from representative growth conditions are presented. (**A**) *ssaQ* under anaerobic shock, (**B**) *sseL* in MEP, and (**C**) *mrcB* in MEP. Called TIS are flagged, and the corresponding translation initiation region of alternative TIS (in orange) is indicated.

We previously employed complementary Ribo-seq and proteomics data to understand the biases inherent in proteomics for the detection of sORF-encoded polypeptides (SEPs) from their encoding sORFs (39), a category that is often under-annotated in current genome annotations (5). More specifically, besides total shotgun proteomics data, we obtained matching Ribo-seq data by use of the translation inhibitors retapamulin (RET) and chloramphenicol (CAM), allowing the determination of ribosome occupancy during the initiation (Ribo-RET) and elongation phases of translation, respectively. These complementary data sets were generated from diverse growth conditions, reflecting various bacterial growth phases and environmental stresses (see Materials and Methods and Fijalkowski et al. [39]). In this study, we repurposed the Ribo-seq data specifically to discover expressed Nt-proteoform pairs. For such pairs, in addition to the commonly observed translation initiation at the annotated translation initiation site (referred to as database-annotated TIS or dbTIS) and corresponding to the annotated protein-coding region, Ribo-RET evidence additionally indicated the occurrence of alternative translation initiation (aTIS). Collectively, these TIS events give rise to the expression of Nt-proteoform pairs. As tools utilizing signal distribution have proven effective in delineating novel, true ORFs (15, 16), and our recently published gene detection pipeline selected the longest ORF in the case of in-frame reading frames, implying a bias against calling of in-frame ORFs (39), we conducted ORF calling on these data sets without imposing any length-based filters, yet adhering to stringent thresholds for ORF identification. This revised ORF calling strategy enabled us to identify a set of 26 curated genes, each encoding a pair of putative N-terminal proteoforms. Particularly for extended proteoforms like SseL^L^ (Fig. 1), continuous elongating translation (Ribo-seq) signal upstream of the annotated TIS can be observed. This makes it challenging to detect independent translation initiation at the annotated TIS when expression levels of the long and annotated proteoforms do not significantly differ. Likewise, an increased intensity of the Ribo-seq signal around initiation (39) does not always serve as a definitive metric for discovering truncated proteoforms, as variations in translation speed (e.g., translation stalling) can also lead to local signal accumulation. These shortcomings can, however, be mitigated using the relatively high specificity of the translation initiation signal (Ribo-RET [39]), previously shown to facilitate the Ribo-seq-assisted discovery of alternative proteoforms, and thus concomitantly the concurrent identification of ORFs encoding multiple Nt-proteoforms.

**Fig 2:**
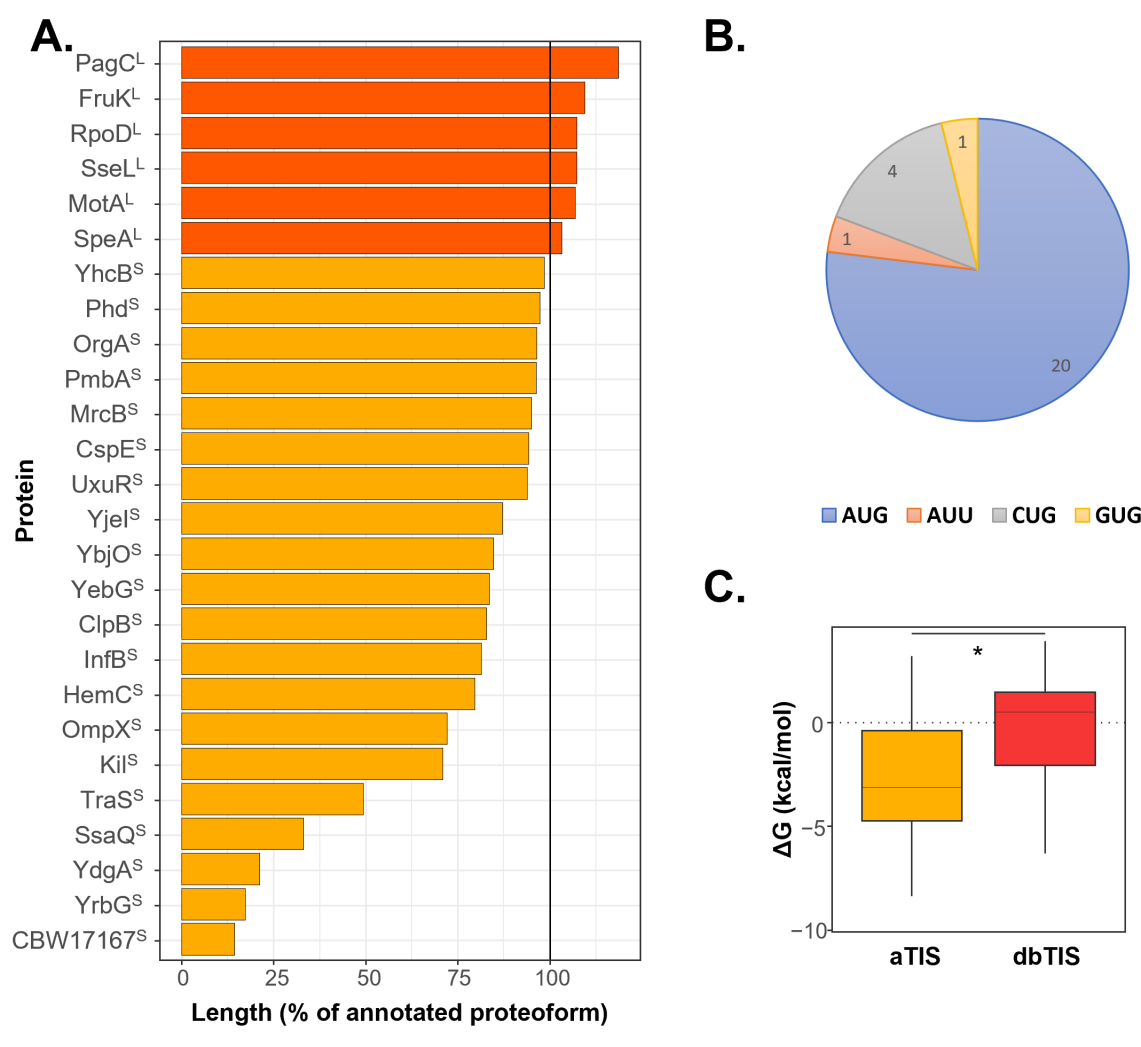
High-confidence N-terminal *S.* Typhimurium proteoform pairs. (**A**) Relationship between the identity and size (expressed as a percentage of the annotated proteoform length) of the newly identified member and annotated proteoform of an N-terminal proteoform pair. N-terminal extensions and N-terminal truncations are represented in dark and light orange, respectively. (**B**) Usage of initiation codons in the newly discovered N-terminal proteoforms. Note that translation initiation at non-near-cognate in-frame, downstream TIS was not considered. (**C**) Shine-Dalgarno (SD)-like context strength for alternative (aTIS) versus database-annotated translation initiation sites (dbTIS) with an asterisk indicating a *P* value < 0.01 (*t*-test). Corresponding free energy predicted values are listed in Table S1.

After manual curation, involving a meticulous inspection of raw and positional reads around the identified TIS using a genome browser (Integrative Genomics Viewer [IGV]) (40) (see Materials and Methods), more than 50% of the called proteoform pairs (50 in total) or 26 proteoform pairs exhibited robust and compelling Ribo-RET signal for both the database-annotated and the alternative translation initiation sites called. The highly confident Nt-proteoform pairs central to this study are depicted in Fig 2A ; Fig S1. This set includes proteoform pairs where the non-annotated member represents either an N-terminal extension (#6) or an N-terminal truncation (#20). Among these novel N-terminal proteoforms, 20 initiated at an alternative AUG start codon, while six initiated at a near-cognate start codon, such as GUG (#1), CUG (#4), or AUU (#1) (Fig. 2B). Thus, approximately one quarter of the newly discovered proteoforms initiates at near-cognate start codons, a higher frequency than observed for annotated TIS, where 88%, 9.2%, and 2.7% initiate at AUG, GUG, and UUG, respectively.

The study by Meydan et al., which explored the alternative translatome of *E. coli*, encompassed 42 genes exhibiting conserved evidence of translation initiation at an in-frame downstream aTIS when comparing two strains (17). In the context of our confident Nt-proteoform pairs, we found evidence for the homologous expression of six proteoforms in our *S. Typhimurium* data sets previously reported in *E. coli* (*mrcB*, *pmbA*, *speA*, *yebG*, *clpB*, and *infB*) (17). Although potential indications for homologous members of additional proteoform pairs reported by Meydan et al. (17) were found in our data set (e.g., *arcB* and *slyB*), the calling of matching aTIS may have been missed by our gene detection tools for various reasons, including low expression of the alternative proteoform (e.g., *arcB*) or the omission of translation initiation at in-frame, downstream non-near-cognate start codons.

### Conservation and experimental proteomic data in support of newly discovered proteoform pairs

**Fig 3:**
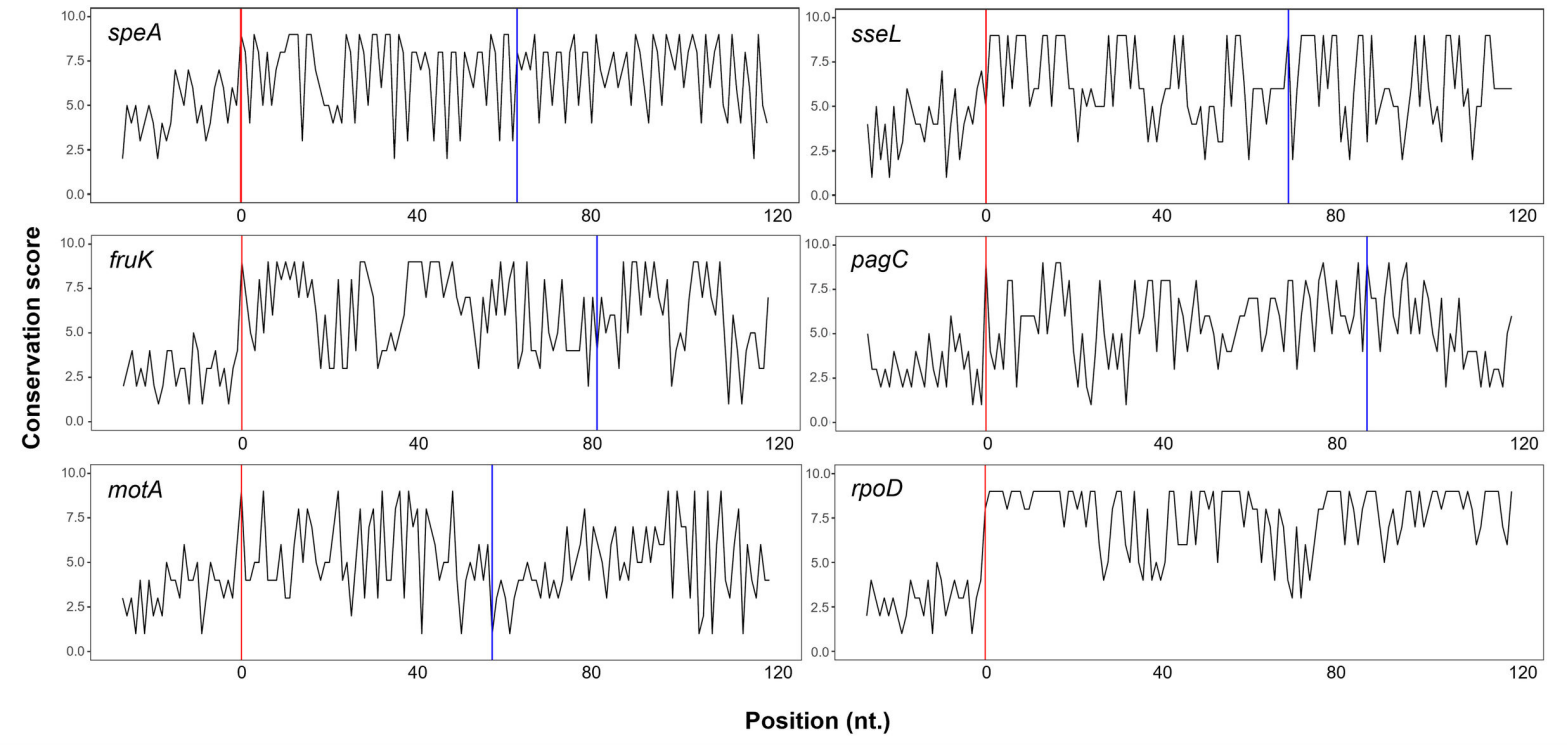
Nucleotide sequence conservation of newly discovered N-terminal extended proteoforms constituting proteoform pairs. Nucleotide sequence conservation remains high throughout the coding sequence encoding the N-terminal extensions (portion between the red and blue lines, with the red and blue lines indicating the first nucleotide [nt.] of the alternative [aTIS] and database-annotated TIS [dbTIS], respectively) of the six Nt-extended proteoforms of proteoform-pair-encoding genes identified, namely *speA*, *sseL*, *fruK*, *pagC*, *motA,* and *rpoD*, as visualized using ConSurf by plotting calculated nucleotide conservation scores (41). Only the first 120 nt. of the annotated coding sequence are shown for simplicity; hence, the missing blue line corresponding to the first nt. matching the dbTIS of *rpoD*.

When comparing the Shine-Dalgarno (SD)-like sequence initiation context, alternative initiation events detected in the context of Nt-proteoform pairs exhibit a significantly stronger initiation context compared to their annotated counterparts (*P* value < 0.01, as detailed in Table S1 and illustrated in Fig. 2C). Additionally, for all six non-annotated extended Nt-proteoform members of identified pairs, the ConSurf calculated conservation scores (41) of the coding sequence remain high throughout the extended sequence of the proteoform beyond the annotated ORF (Fig. 3). The ConSurf analysis involved a standard approach of homologous gene search for each candidate proteoform, followed by conservation scoring using the Rate4Site algorithm and subsequent phylogenetic tree reconstruction, as previously described (41). Moreover, (homology-inferred) UniProt database (unreviewed) entries for all six non-annotated extensions were identified in SL1344 and/or related LT2 and 14028s strains (e.g., SseL^L^ [e.g., A0A719CWE6], SpeA^L^ [A0A719A111], FruK^L^ [Q7CQ78], PagC^L^ [P23988], MotA^L^ [A0A718V5M5], and RpoD^L^ [A0A0F6B6Z4]). These findings support the true translation initiation and coding potential of the Nt-extended proteoforms identified by Ribo-RET.

**Fig 4:**
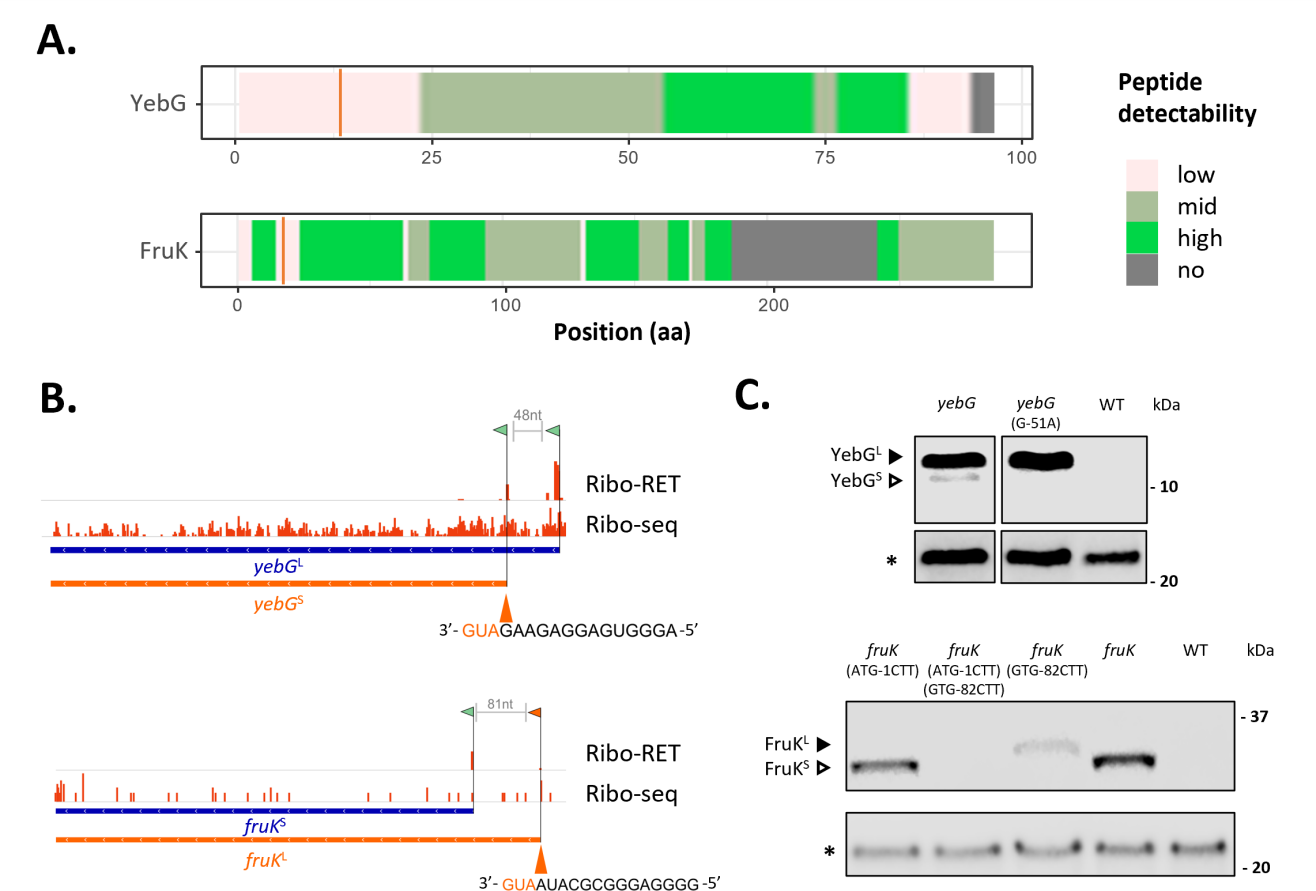
Riboproteogenomic identification and blotting-based validation of the expression of YebG and FruK *S*. Typhimurium Nt-proteoform pairs. (**A**) AP3-derived detectability scores (43) of the YebG^L^ and FruK^L^ proteoforms (with the small proteoform region past the orange line). Peptide detectability is represented in a color code according to the AP3 scale: high detectability (scores greater than 0.9) is indicated in green, medium detectability (scores between 0.4 and 0.9) in light green, and low detectability (scores less than 0.4) in pale red. (**B**) Ribo-RET (RET) and Ribo-seq signal for *yebG* and *fruK* in salt shock (NaCl) is shown. Called TIS are flagged, and the corresponding translation initiation region of the alternative TIS (orange) is indicated. (**C**) HiBiT blotting of corresponding *S*. Typhimurium SL1344 wild-type, *yebG::HiBiT Km^R^* and *yebG*^G-51A^*::HiBiT Km^R^* (Chr: 1,933,051 G > A), *fruK^ATG-1CTT^::HiBiT Km^R^* (Chr: 2,303,857 – Chr: 2,303,859 ATG > CTT), *fruK^GTG-82CTT^::HiBiT Km^R^* (Chr: 2,303,857 – Chr: 2,303,859 GTG > CTT), and *fruK^ATG-1CTT,GTG-82CTT^::HiBiT Cm^R^* and *fruK::HiBiT Km^R^* protein extracts (ESP, OD600 2.0 cultures) using chemiluminescence detection validates the (selective) expression of the YebG and FruK proteoforms. Arrows indicate sizes of corresponding large (black arrow) and small (white arrow) Nt-proteoform members (YebG^L^ [12.0 kDa], YebG^S^ [10.2 kDa], FruK^L^ [35.0 kDa], and FruK^S^ [32.0 kDa]). (*) A band detected by streptavidin blotting (S680) and corresponding to an endogenously biotinylated *S*. Typhimurium protein served as loading control.

As ribosomal occupation does not always relate to genuine protein production (42), experimental mass-spectrometry (MS)-based detection is a common go-to method to validate newly discovered translation events inferred from Ribo-seq. Initially, the theoretical MS detectability of Nt-proteoform indicative tryptic peptides was assessed using the advanced proteolytic peptide predictor algorithm AP3 (43) (Fig. S2). For univocally identifying proteoform members constituting a proteoform pair, and because the vast majority of their identifiable peptides are shared (i.e., the peptide coverage is on average over 90% identical when considering the two members of the Nt-proteoform pairs identified here), Nt-peptides may often serve as the sole means of confident Nt-proteoform detection by acting as proxies of translation initiation. Our analyses highlight that finding unique proteomic support is challenged by the scarcity of MS-detectible proteoform-specific tryptic peptides. Specifically, in the specific case of the YebG^L^, the theoretical N-terminal peptide detectability is very low, making direct and univocal MS detection of YebG^L^ unlikely (Fig. 4A).

Nonetheless, to explore riboproteogenomic matching support for our confident list of Nt-proteoform pairs, complementary proteomics data were examined (15, 16, 22). When interrogating Ribo-seq matching shotgun and N-terminal proteomics data, four translation initiation-indicative Nt-peptides (Nt-peptides of RpoD^S^, CspE^L^, and YebG^L^) were found. Besides, proteogenomic peptides exclusively mapped to the unique extensions of PagC^L^, FruK^L^, SpeA^L^, PmbA^L^, MrcB^L^, OmpX^L^, HemC^L^, ClpB^L^, InfB^L^, YdgA^L^, and SL1344_1071^L^ (Table S1) were identified. Collectively, proteomic support for 14 proteoform members of the 26 confident Nt-proteoform pairs has been discovered. Of note, complementary data for the expression of the homologous PagC and OmpX proteoform pairs come from the observation that annotated OmpX^L^ and newly discovered PagC^L^ proteoform sequences display 37% sequence identity (Clustal O alignment, v1.2.4), with conservation of the TIS matching the extended proteoforms.

**Fig 5:**
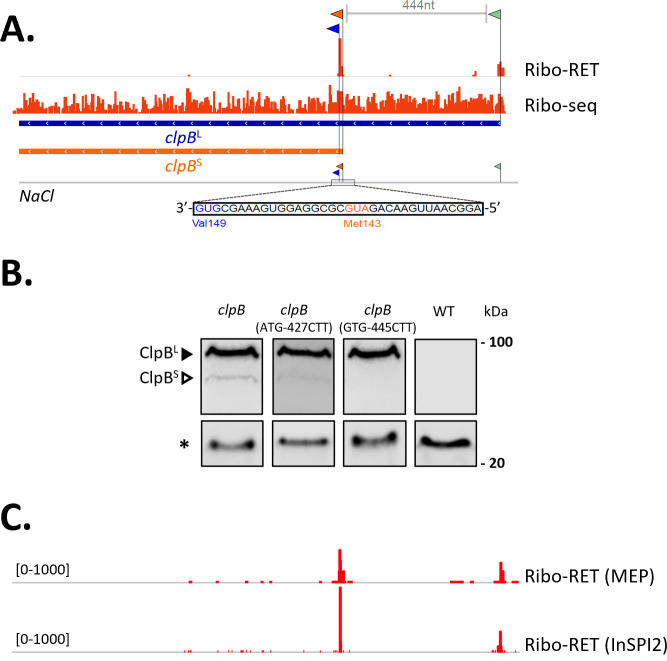
Riboproteogenomic identification and blotting-based validation of the expression ClpB Nt-proteoform pairs in *S*. Typhimurium under regulatory control. (**A**) Ribo-RET (RET) and Ribo-seq signal for *clpB* in salt shock (NaCl) is shown. Called TIS are flagged, and the corresponding translation initiation region of the alternative TIS (corresponding to Met143) is indicated (orange). The previously confirmed aTIS of ClpB^S^, corresponding to Val149, is flagged in blue. (**B**) HiBiT blotting of corresponding *S*. Typhimurium SL1344 wild-type, *clpB::HiBiT Km^R^, clpB^ATG-427CTT^::HiBiT Km^R^* (Chr: 2,804,674 – Chr: 2,804,676 GTG > CTT), and *clpB*^GTG-445CTT^*::HiBiT Km^R^* (Chr: 2,804,656 – Chr: 2,804,658 GTG > CTT) protein extracts (ESP, OD600 2.0 cultures) using chemiluminescence detection validates the (selective) expression of the ClpB proteoform pair. Arrows indicate sizes of corresponding large (black arrow) and small (white arrow) Nt-proteoform members (ClpB^L^ [96.7 kDa], ClpB^S^ [81.0 kDa]). (*) A band detected by streptavidin blotting (S680) and corresponding to an endogenously biotinylated *S*. Typhimurium protein served as loading control. (**C**) Ribo-RET tracks of *clpB* shown in the same scales reveal differential expression of the two proteoforms when comparing MEP versus *Salmonella* pathogenicity island 2-inducing (InSPI2) growth conditions.

To obtain complementary evidence of Nt-proteoform pair expression, especially in cases where finding MS-supportive evidence is unlikely (e.g., YebG, Fig. 4A), we pursued the validation of endogenous expression for the YebG, ClpB, and FruK proteoform pairs using HiBiT blotting (44). Ribo-RET evidence suggested translation initiation from the annotated dbTIS (AUG) and initiation at a downstream in-frame AUG for all three cases (Fig. 4B and 5A). The small luminescent peptide tag HiBiT was chromosomally introduced as a C-terminal tag through recombineering, allowing for sensitive detection of endogenous protein expression. In the case of HiBiT-tagged YebG and ClpB in control cells, two distinct molecular weight (MW) bands were observed—with the lower band being fourfold less intense in the case of *yebG* and ninefold less in the case of *clpB*—at the corresponding molecular weights of the YebG and ClpB proteoforms (Fig. 4C and 5B). For FruK—likely due (in part) to the relatively small molecular weight difference (3 kDa) and/or the difference in expression of the two FruK proteoforms—no two discrete bands were apparent (Fig. 4C). Endogenous site-specific mutagenesis by means of oligo-mediated allelic replacement (OMAR) confirmed that the lower MW band of HiBiT-tagged YebG corresponds to YebG^S^ (45). Specifically, the Ribo-RET-called ATG aTIS (encoding M17) was mutated to ATA (encoding I17) resulting in the exclusive expression of YebG^L^ (Fig. 4C). Interestingly, mutating the Ribo-RET-called GTG dbTIS (encoding M27) matching FruK^S^ to the non-near-cognate start codon CTT did not abolish FruK expression overall but resulted in a fivefold expression reduction, indicative of the exclusive expression of the newly discovered FruK^L^ and the expression of FruK^L^ at lower levels—especially in control cells—as compared to FruK^S^, in line with the corresponding Ribo-RET data (Fig. 4B). This was also apparent from the slightly higher MW band over the (composite) band detected in the *fruk::HiBiT* control strain. Mutation of both TIS completely abolished FruK expression. Although we have obtained conclusive support for the expression of N-terminal (Nt)-proteoform pairs, some validation efforts highlight the need for further refinement in the resolution of TIS calling. Specifically, in the case of ClpB (ClpB^S^), we identified an aTIS corresponding to Met143. However, mutating this *clpB* aTIS still resulted in the expression of a ClpB proteoform pair. In line with the previously experimentally confirmed aTIS of ClpB corresponding to Val149 (46), it was only when mutating this corresponding GTG TIS to CTT that exclusive expression of ClpB^L^ was observed, confirming the true nature of this TIS (Fig. 5B).

### Ribo-RET peak intensity can serve as a proxy for Nt-proteoform expression

Although the general read distribution in ribosome profiling data sets is non-uniform and exhibits 5’ and 3’ polarization, especially in case of bacterial Ribo-seq data sets (11, 13, 47), these data sets have been successfully used for accurate protein synthesis quantification (13). However, to ensure unbiased analysis, the outer 5’ and 3’ codon occupancies of CDSs must be trimmed, preventing the inherent increased read density at these positions from influencing the analysis (14). Accumulation at the 5’ of the coding sequence originates from the fact that while elongation is effectively blocked, initiation ensues, causing ribosome accumulation at the start. Due to the substantial overlap in their sequences, quantifying Nt-proteoform expression of expressed Nt-proteoform pairs is complicated and has been unexplored thus far. However, as evident from the metagene plots displaying typical read distribution in Ribo-seq and Ribo-RET samples, and with the narrow initiation peaks of Ribo-RET data denoting effective translation initiation (39), Ribo-RET data do not suffer from the constraints observed in the case of elongating Ribo-seq data when distinguishing overlapping ORFs. This property makes Ribo-RET data potentially suitable for estimating translation in the case of overlapping or dual-encoding ORFs, as previously demonstrated for analogous lactimidomycin data in eukaryotes (47).

**Fig 6:**
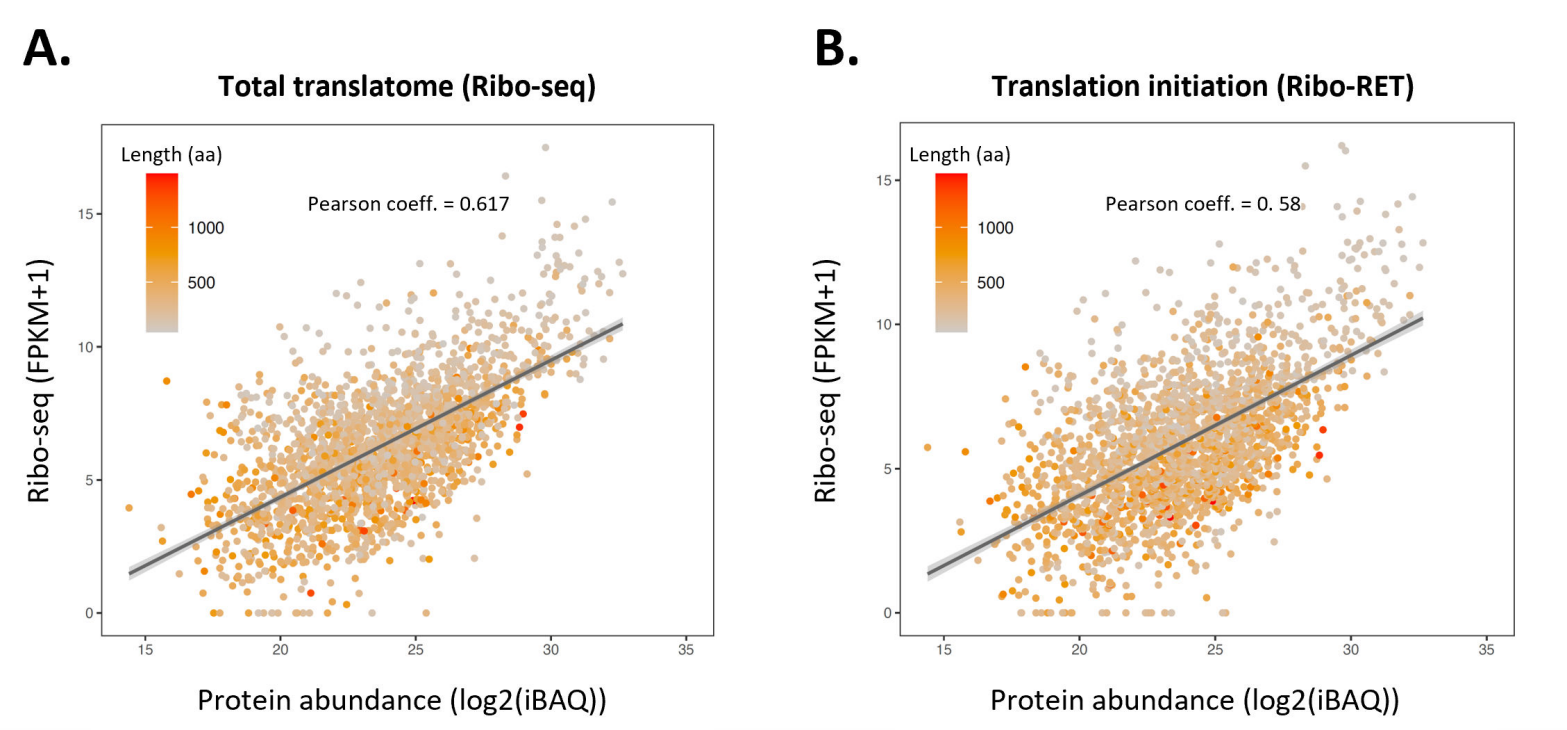
Quantifying protein expression using Ribo-seq and Ribo-RET data. (**A**) Correlation between Ribo-seq determined translation measure and protein steady-state abundance (iBAQ) measured by proteomics in the matching MEP growth condition. (**B**) Correlation between Ribo-RET determined translation initiation measures and protein steady-state abundance (iBAQ) measured by proteomics in the matching growth conditions (MEP). Analysis has been performed for 3,027 annotated *S*. Typhimurium protein identifications (39).

Interestingly, by correlating Ribo-seq and Ribo-RET data sets with matching protein abundance estimates obtained through proteomics, we demonstrate that Ribo-seq and Ribo-RET data correlate with proteomics-determined protein abundances to a similar extent. For instance, as illustrated for the MEP condition, the Pearson coefficient is 0.58 for Ribo-seq and 0.617 for Ribo-RET, respectively (Fig. 6A and B). This indicates that the Ribo-RET signal serves as a quantitative measure of translation and can therefore be exploited for the quantification of protein expression. This property is of particular interest in the context of quantifying N-terminal proteoform expression, as well as the quantification of translation products originating from overlapping CDSs and sORFs.

### Differential expression of proteoforms

**Fig 7:**
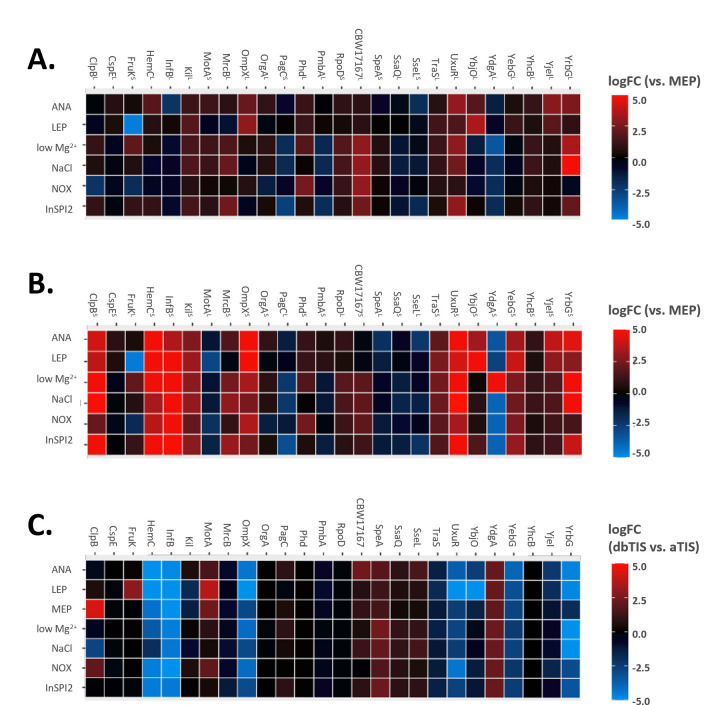
Expression of the members of N-terminal proteoform pairs based on Ribo-RET across a series of growth conditions. (**A**) Expression of annotated proteoforms across investigated growth conditions as compared to expression in MEP. (**B**) Expression of alternative proteoforms across investigated growth conditions as compared to MEP. (**C**) Expression ratio between the alternative and the annotated proteoform members of proteoform pairs across investigated growth conditions. ANA, anaerobic growth; LEP, late exponential phase; InSPI2, SPI2-inducing conditions (growth in low-pH PCN medium); low Mg^2+^, InSPI2 growth in low magnesium containing PCN.

The ability to measure proteoform expression effectively within and between biological conditions lays at the foundation for building a functional understanding of their biological relevance. Leveraging the quantitative aspect of Ribo-RET signal intensity, we quantified the expression of Nt-proteoforms across the investigated growth conditions. This approach allowed us to explore differential Nt-proteoform expression, providing an initial insight into their (conditional) dependency of expression (Fig. 7).

Interestingly, besides the condition-specific expression patterns observed for some genes encoding multiple proteoforms (Table S1), we additionally noted that individual members of certain proteoform pairs exhibit varying expression ratios across different growth conditions. While the origin—and thus expression—of alternative N-terminal proteoforms can potentially be explained by the expression of alternative transcripts, the analysis of complementary RNA sequencing data (16) combined with the review of previously published efforts to map transcriptional start sites (TSSs) in the *S.* Typhimurium SL1344 strain (48) indicate that for 25 out of the 26 N-terminal proteoforms under study, both the annotated as well as the alternative translation initiation sites are situated within annotated or experimentally detected transcripts (Table S1). Therefore, differential expression of the Nt-proteoform pairs under study is likely primarily explained at the level of translation and suggests that the selection of dbTIS versus aTIS, along with the corresponding expressed proteoforms, is subject to differential translational control. Intriguingly, in the case of the ClpB, YrbG, and, YdgA proteoform pairs, the expression of a specific proteoform member was notably strongly favored under infection-relevant (InSPI2) conditions (Table S1 ; Fig. S5C). This observation aligns with previous experimental findings during heat shock in *E. coli*, where ClpB proteoforms similarly showed an increased ClpB^S^:ClpB^L^ expression ratio under thermal stress conditions (49).

### Potential influence on protein localization and physiochemical properties of alternative N-terminal sequences

Using available metadata of annotated proteoforms, including EffectiveDB (50) and PSORTb (51) predicted protein localizations, we identified seven genes (*sseL*, *ssaQ*, *ydgA*, *yfhG*, *mgtC*, *ompX*, and *pagC*) that could potentially exhibit distinct subcellular localizations among their constituting Nt-proteoform members. In the case of the type III effector (T3E) deubiquitinase SseL, the newly identified 23-amino acid extended proteoform SseL^L^ is predicted to contain a T3E secretion signal in its N-terminal extension (EffectiveT3 model 2.0.1, score 1.0). This signal promotes secretion/translocation of theT3E SseL^L^ proteoform via the T3SS. Consequently, annotated SseL^S^ is likely to remain localized in the bacterial cytosol due to the absence of this signal peptide. Supporting this observation, Niemann et al. reported the translocation of SseL fused to a Cya reporter only when including a part of its so-called 5′ UTR (52). Similarly, the *Salmonella* pathogenicity island 2 (SPI2)-encoded T3SS protein SsaQ^L^ localizes to the basal body of the flagellum, while SsaQ^S^ is predicted to reside in the cytoplasm, potentially playing a role in T3SS assembly similar to its homolog SpaO (53). Furthermore, the truncated version of the lipoprotein encoded by *yfhG* may lose its outer membrane localization, and MgtC^S^ might not be (efficiently) integrated within the membrane compared to their longer counterparts. Lastly, the truncated DNA topoisomerase YdgA^S^ is predicted to lose its membrane attachment.

**Fig 8:**
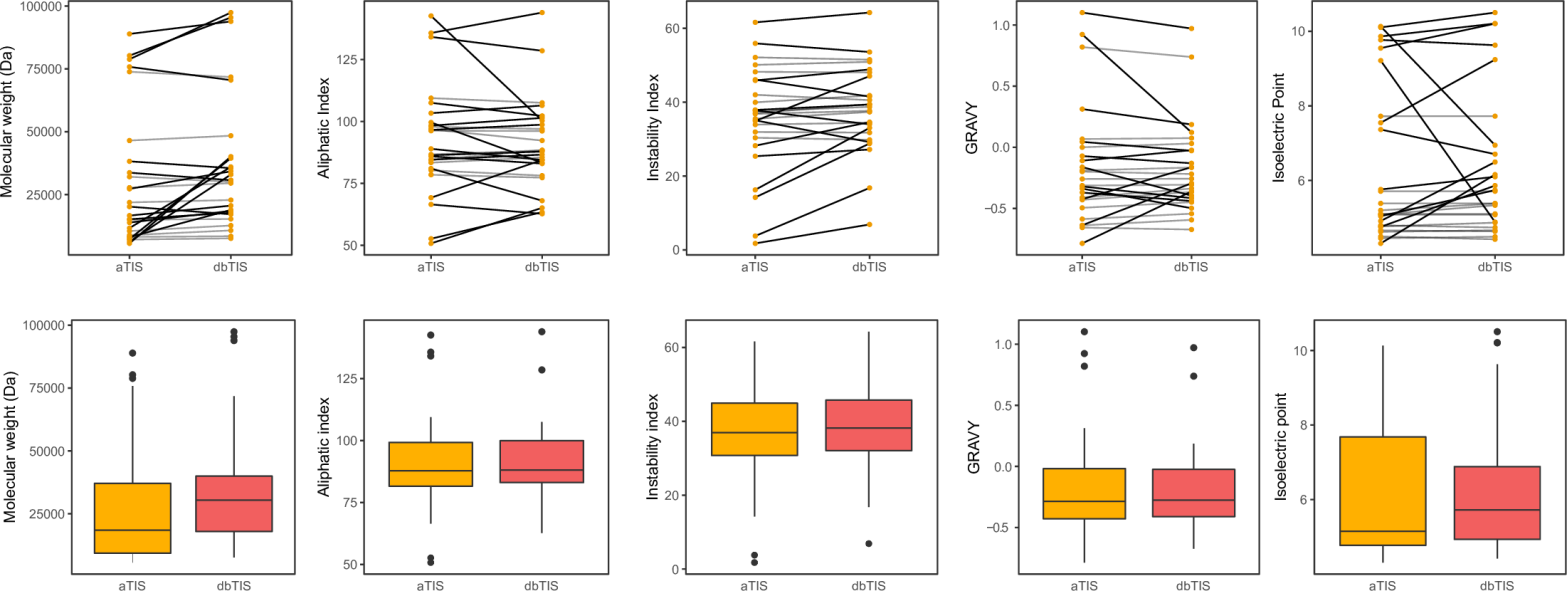
Physiochemical properties for alternative and annotated forms of Nt-proteoform pairs may differ. Molecular weight (MW, Da), aliphatic index, instability index, GRAVY hydrophobicity score, and isoelectric point of alternative (matching aTIS, dark yellow) and annotated (matching dbTIS, red) proteoforms are presented. Lines in the line plots (upper panels) are in black only when differences between the members of a pair are significant (*t*-test, *P* value < 0.01) (Table S2).

Furthermore, we computed a series of physiochemical properties for both the alternative and the annotated forms of the 26 proteoform pairs identified in this study (Table S2; Fig. 8). Despite the low average impact on protein length (Fig. 1A), with only five proteoforms (TraS, SsaQ, YdgA, YrbG, and CBW17167) showing a reduction of over 50% in length, differences in N-terminal sequences can lead to altered proteoform properties, as (localized) sequence characteristics contained within these N-terminal sequences can vary considerably (Table S2; Fig. 8).

**Fig 9:**
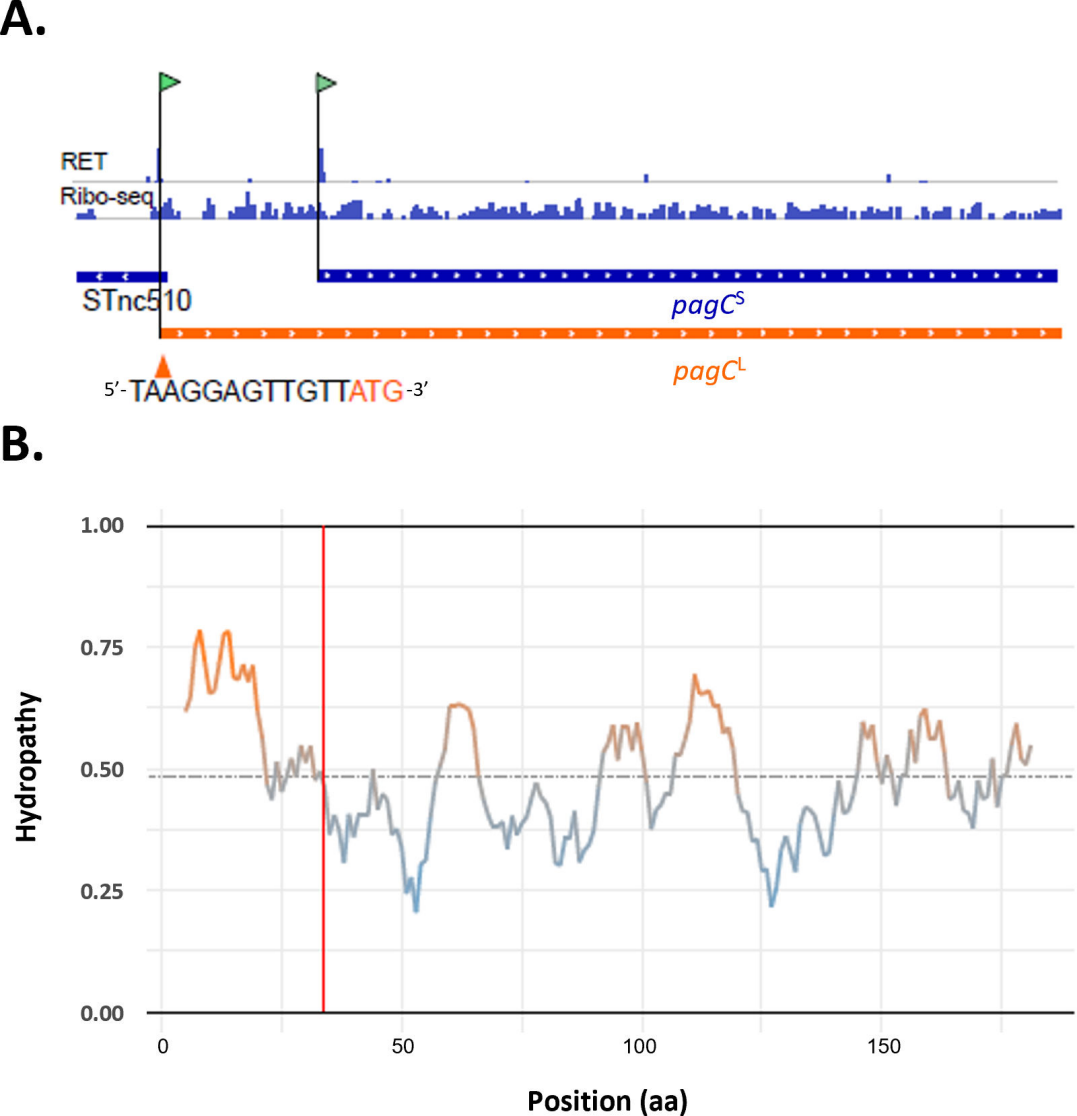
Identification and characterization of PagC proteoforms. (**A**) Matching Ribo-RET and Ribo-seq evidence indicative of the translation of PagC Nt-proteoform pairs in *S*. Typhimurium. Ribo-seq signal of late exponential growth phase (LEP) is shown for *pagC*. (**B**) Differing amino acid sequence properties between extended (PagC^L^) and annotated (PagC^S^, sequence beyond the vertical red line) PagC proteoforms are illustrated here in the hydropathy plot.

As an illustrative example, the 29-amino acid extension of PagC^L^ (Fig. 9A) exhibits a higher scaled hydropathy when compared to its annotated counterpart (Fig. 9B). SignalP 6.0 (54) predicts a Sec signal peptide with cleavage predicted between Ala23 and Asp24 in PagC^L^, resulting in the generation of a proteolytic PagC proteoform with N-terminal proteomics support (i.e., the neo-N-terminus D_24_TNAFSVGYAQSKVQDFKNIR was identified by means of N-terminal proteomics) (15, 39). Similarly, for homologous OmpX, a Sec signal peptide was predicted spanning amino acid sequences 1–23. Consequently, the annotated PagC^S^ and the newly identified OmpX^S^ proteoforms both lack this signal peptide and cleavage site, potentially leading to differential subcellular localization of these truncated proteoforms.

## DISCUSSION

N-terminal proteoforms have been identified across a diverse range of species, spanning humans, rodents, plants, viruses, and bacteria (10, 14, 25, 55, 56). Albeit relatively uncharted in case of bacteria, the exploration of bacterial proteoforms that underwent detailed functional characterization has proven to play crucial roles in essential aspects of bacterial physiology, as recently reviewed (10, 26). From the essential coexpression of two SsaQ proteoforms in *Salmonella*, also identified in our study, facilitating effective infection (31), to the differential regulation of phosphorylation by CheA proteoforms in *E. coli* (33), N-terminal proteoforms arising from (alternative) translation initiation events have been shown to fulfill distinct functional roles.

Bacterial genome-wide translatome studies and, particularly, translation initiation profiling (Ribo-RET) offer a means to comprehensively detect bacterial proteoforms of translational origin (17). However, existing tools for genome (re-)annotation using translatomics data face challenges in Nt-proteoform discovery, often favoring longer predictions (15, 16). In light of recent advances in genome (re-)annotation efforts exposing the underappreciated complexity of bacterial genomes (4, 6, 21, 55, 57–60), studying the selective expression of N-terminal proteoforms represents a crucial step toward a better understanding of bacterial proteoform biology. In this study, we confidently identified 26 N-terminal proteoform pairs in the model bacterial pathogen *S. Typhimurium*, conducted *in silico* characterization, and validated proteoform expression using proteomics and HiBiT blotting-based detection. Furthermore, we demonstrate for the first time that translation initiation metrics can be used to effectively quantify proteoform expression across biological conditions, allowing for their functional characterization while providing the first (differential) expression atlas of N-terminal proteoforms in bacteria to date. Despite the substantial overlap between homologous proteoforms detected in the current and previous landmark *E. coli* study (17), observed discrepancies, especially in TIS calling resolution (e.g., the previously reported and experimentally confirmed aTIS of *clpB* corresponding to Val149 [46] while we called an aTIS corresponding to Met143 [Table S1 ; Fig. 5]), suggest the need for further improvements in gene detection algorithms to fully utilize the potential of Ribo-RET data sets especially since rigorous determination of the initiation triplet is critical for future TIS mutagenesis studies. Although increased resolution in TIS calling may be anticipated with recent advances in computational technology and deep learning (61), conservation analysis or N-terminomics efforts could also contribute to TIS refinements.

On the other hand, technical constrains persist in bacterial ribosome profiling techniques due to the use of more sequence-biased nucleases (MNase), resulting in inconsistent footprint lengths and more error-prone P-site assignment, lowering data resolution compared to its eukaryotic counterpart RNase I. In practical terms, ribosomal footprint coverage of multiple, neighboring (near-cognate) initiation codons can complicate confident delineation of the selected TIS and, consequently, correct TIS assignment. Moreover, the effective proteomics validation of the discriminative N-terminal part of proteoforms is challenging due to the scarcity of MS-detectable peptides (Fig. 4A; Fig. S2) (43), presenting a major hurdle that should be addressed by future technological advances. Currently, targeted proteomics strategies employing actinonin treatment combined with N-terminomics hold the highest potential for effective proteome-wide scale validation efforts of bacterial Nt-proteoform expression at single amino acid resolution (30, 62).

At an individual level, TIS mutagenesis proves effective for steering selective proteoform expression, opening up possibilities for differential functional proteoform studies, including interactomics (30) and phenotypic analysis (e.g., impact on virulence [31]). We demonstrate, for the first time, that steering and monitoring the endogenous expression of individual proteoforms can be accomplished effectively using multiplexed recombineering (i.e., ssDNA oligo and dsDNA-based recombineering). Scar-free TIS mutagenesis through OMAR (45) and concomitant introduction of a protein tag (e.g., HiBiT [44]) integrated in a selectable dsDNA cassette was successfully performed using multiplexed recombineering approaches (44, 63) (Fig. 4 and 5).

Nonetheless, validating the expression of Nt-proteoform pairs poses considerable challenges. These challenges arise due to frequently subtle differences in molecular weights, which can be attributed to closely spaced TIS (e.g., YebG) or post-translational proteolytic processing of specific proteoform members (e.g., PagC), or simply by significant disparities in proteoform abundance (e.g., FruK). Moreover, the impact of mutation introduced at the TIS on potential (efficiency of) alternative translation initiation cannot be overlooked, as the usage of alternative start sites has been reported in such scenarios (64) and also shown here in the case of FruK (Fig. 4C).

Despite the challenging nature of distinguishing Nt-proteoform identities using proteomic means, recent developments in proteome-wide localization studies allow, at least for the subset of detectable variants, the study of their differential localization (65). A simplified differential ultracentrifugation protocol could possibly shed more light on the (differential) localization of proteoforms at a proteome-wide scale. Moreover, similar to human proteoform efforts, investigating the potential impact on proteoform stability could provide valuable insights (25). Given the overall robust expression levels of alternative proteoforms detected in this study, their regulation, including unraveling the mechanisms driving alternative proteoform selection, remains an interesting avenue for future research, especially considering the absence of leaky scanning in prokaryotes. This exploration will aid to elucidate the functional implications of alternative transcription, alternative translation initiation, and potentially the action of alternative ribosomes (66) on the (selective) expression of alternative proteoforms, as well as the coordination of their functioning.

In addition to the themes outlined above, our findings unveil a notable enrichment for genes encoding proteoform pairs among (inner membrane) proteins that are predisposed to form higher-order homo- or hetero-oligomeric structures (e.g., FruK, ClpB, MotA, MrcB). Particularly, the MrcB proteoform pair is marked by a 42-amino acid extension in the longer variant preceding the transmembrane anchor and comprising a disordered N-terminal region enriched with a basic stretch of lysine and arginine, followed by an acidic sequence dominated by glutamate and aspartate residues. Notwithstanding the proven identical enzymatic functions of MrcB proteoforms, this unique polar region points to alternative modes of membrane interaction as shown for its homologous *E. coli* proteoform (26, 67, 68). Similarly, outer membrane β-barrel proteins like OmpX and PagC exhibit an interesting proteoform dichotomy; their shorter proteoform counterparts lack the signal peptide present in the longer forms, leading to a mature proteoform length that may be indistinguishable from the short proteoform due to post-translational signal processing. Furthermore, a subset of proteoform-encoding genes, such as SsaQ (and related SpaO), OrgA, and SseL, is implicated in T3E secretion and tends to exhibit conservation that is largely restricted to the *Salmonella* genus, highlighting their specialized roles in pathogenicity. Intriguingly, our proteoform-mapping effort also spotlights the *kil*/*phd* toxin/antitoxin system, wherein the two members encoded within the same cistronic unit showcase expression of proteoform pairs, pointing to a nuanced regulatory mechanism at play within this toxin–antitoxin interaction.

The expanding catalog of bacterial proteoforms of translational origin necessitates future validation and functional characterization efforts. Much like genome (re-)annotation efforts in general, effective detection of bacterial N-terminal proteoforms lays the foundation for such future studies. Using the workflow presented in this study, N-terminal proteoforms can be detected in other bacterial species, as previously shown in *E. coli* (17), thereby providing more comprehensive pictures of (conditional) translation initiation landscapes while aiding our understanding of bacterial proteoform biology. Given that various proteins involved in bacterial pathogenicity display alternative Nt-proteoform expression, gaining insights on their biological function can moreover enhance our understanding of bacterial infections.

## MATERIALS AND METHODS

### Bacterial culture conditions used for riboproteogenomics data analysis

The *S. enterica* serovar Typhimurium wild-type (WT) strain SL1344 (69) (genotype: hisG46, phenotype: His(-); biotype 26i) was acquired from the *Salmonella* Genetic Stock Center (SGSC, Calgary, Canada; cat# 438 [69]). Bacterial growth conditions, as previously detailed by Fijalkowski et al. (39), were performed in liquid Lennox broth (LB) growth medium (10 g/L Bacto tryptone, 5 g/L Bacto yeast extract, 5 g/L NaCl) or various formulations of phosphate carbon nitrogen (PCN) medium (70) (InSPI2; *Salmonella* pathogenicity island 2-inducing condition; pH 5.8, 0.4 mM Pi), as described previously (39). These conditions encompassed early exponential growth phase (EEP; OD600 0.1), mid-exponential growth phase (MEP; OD600 0.3), late exponential growth phase (LEP; OD600 1.0), early stationary phase (ESP, OD600 2.0), and late stationary phase (LSP, OD600 2.0 + 6 h of extra growth). Additionally, bacterial cultures at MEP were subjected to osmotic shock (0.3 M NaCl shock for 10 min), anaerobic shock, SPI2-inducing PCN (InSPI2; pH5.8, 0.4 mM Pi), low-magnesium SPI2-inducing PCN (low Mg^2+^; pH5.8, 0.4 mM Pi) containing low levels (10 µM) of magnesium sulfate or MEP-grown InSPI2 cultures subjected to nitric oxide shock (addition of Spermine NONOate to a final concentration of 250 µM for 20 min). All PCN media were supplemented with 5 mM final concentration (f.c.) histidine. These diverse growth conditions were chosen to capture a comprehensive snapshot of the riboproteogenomic landscape of *S*. Typhimurium. Ribosome profiling and Ribo-RET analyses were performed in biological duplicates as described by Fijalkowski et al. (39).

### Plasmids and oligos

The pORTMAGE-2 helper plasmid (71) obtained from Addgene (plasmid #72677) encodes Gam, Beta, and Exo, in addition to a dominant negative *mutL* (E32K) allele. The expression of these elements is collectively controlled by the temperature-sensitive cl857 repressor. All oligonucleotides utilized in this study are detailed in Table S3 and were procured from Integrated DNA Technologies (IDT, Leuven, Belgium). Standard desalted purification was applied to 25 nmol oligos, while high-performance liquid chromatography (HPLC) purification was implemented for 100 nmol modified oligos (IDRT, Coralville, IA, USA). The oligos were resuspended in water, achieving a final concentration of 100 µM. Single-stranded DNA (ssDNA) oligos used for OMAR were designed using the Mage Oligo Design Tool (MODEST) (72). Modified ssDNA oligos, featuring two phosphorothioate linkages between the ultimate 5’ and 3’ nucleotides, and occasionally incorporating a 2-fluoro-uridine modified base within the range of five bases around the mismatched nucleotide responsible for the introduction of the point mutation, were synthetized for TIS mutagenesis.

### Ribo-RET data analysis

Sequencing files underwent demultiplexing through the bcl2fastq software (Illumina). Sample-specific fastq.gz files, originating from individual lanes, were concatenated using Unix’s “cat” command. NextFlex library-introduced Unique molecular identifiers (UMI) were extracted from individual reads using a custom Python script. PCR bias normalization was performed using a previously established normalization procedure (47). Normalized data underwent a two-step trimming process using cutadapt. The first step involved the removal of standard Illumina adapters (cutadapt -q 20 -m 25 -e 0.2), followed by the secondary removal of UMIs (cutadapt -u 4 -u -4). Data were mapped to indexed ribosomal RNA (rRNA) sequences using Bowtie (bowtie -t -n 2 -p 6 –best). rRNA sequences retrieved from Ensembl and Genebank were supplemented with additional sequences of tRNAs, RNA subunits of nucleoproteins, and non-coding RNAs (ncRNAs) to the rRNA index. Reads aligning to these sequences were excluded from further analysis. The remaining reads were mapped to the *S. Typhimurium* SL1344 genome (Ensembl, GCA_000210855.2) using Bowtie (-t -n 2 -p 6 -m 1 --best --strata –sam). The resulting SAM files were converted to BAM format and were sorted using Samtools (73). RiboWalz (74), recognized for superior performance compared to plastid (75) used in previous studies (16), was employed for ribosomal P-site assignment for all reads. Positional data were counted using a custom Python script, normalized to reads per million (RPM) values, and were further processed for the creation of positional read occupancy tables in Python and R tidyverse environment. The Reads Per Kilobase of transcript per Million reads mapped (RPKM) values were then calculated for all Ensembl annotated genes. Separate BedGRaph files were generated using a custom Python script for effective data visualization, as detailed by Fijalkowski et al. (39). Sequencing statistics and metagene analysis has been reported by Fijalkowski et al. (39).

### Ribo-seq inferred ORF detection and quantification of proteoform translation

Detailed procedures for the generation and sequencing (statistics) of ribosome profiling libraries of *S*. *Typhimurium* SL1344 libraries have been described previously (39). ORF delineation from Ribo-RET was essentially performed as described by Fijalkowski et al. (39). Notably, data filtration was omitted, allowing the unbiased selection of multiple in-frame ORFs and thus multiple alternative translation initiation events per ORF. Sliding window signal detection was executed using a custom Python script. A global average coverage per codon served as the background cutoff, subtracted from each genomic position before retapamulin signal detection. This ORF calling strategy yielded a set of 50 putative N-terminal proteoform pairs. The candidates were manually curated, involving detailed inspection of raw and positional reads surrounding the identified TIS for evidence of robust (>5 RPM) initiation signal clearly distinguished from surrounding background signal, precise alignment of raw read center with the putative TIS (within 9nt window from Ribo-Walz [74] inferred ribosomal P-site) and overall sequencing quality (sequencing quality score >30 and mapping quality score MAPQ > 30, including only uniquely mapped reads). Following this curation, over 50% (26) high-confidence proteoform pairs were retained. The curated high-confidence ORF list underwent further processing using custom R and Python scripts, as previously detailed (39). A differential expression analysis for ORFs detected across all investigated Ribo-seq conditions was conducted using the DESeq R package (76). Corrected retapamulin intensity values used in the expression analysis were derived as the integral of the intensity of the retapamulin peak detected normalized against the detected average expression intensity within the body of the gene (background).

### Conservation analysis

We conducted a comprehensive evolutionary conservation analysis of the alternative proteoforms using the ConSurf pipeline (41). Initially, the nucleotide sequences of the putative proteoforms were BLAST-searched against the UNIREF-90 database to identify homologous sequences. Redundant homologous sequences were excluded, and the remaining sequences were aligned using MAFFT, facilitating the reconstruction of a phylogenetic tree. The phylogenetic tree, alongside calculated evolutionary distances and the multiple sequence alignment, was then utilized in the Rate4Site algorithm allowing for Bayesian calculation of evolutionary rates. After normalization and binning, the conservation scores were determined on a scale from 1 (indicating high variability) to 9 (denoting strong evolutionary conservation) (Fig. 3). For truncated proteoforms, where the alternative start codon is embedded within a generally highly conserved coding sequence, we assessed the conservation of the start codon and its associated Shine-Dalgarno context across 100 genomes. These genomes are representative of all major branches in the Enterobacteriaceae phylogeny, including the 10 most complete genomes from *Escherichia, Shigella, Salmonella, Citrobacter, Enterobacter, Klebsiella, Erwinia, Serratia, Yersinia, Morganella*, *and Proteus*, as available in the NCBI database. The genome sequences were aligned using ClustalW (v.2.1). The conservation of the start codon and its associated Shine-Dalgarno sequence was categorized into three groups: sequences (partially) conserved within the *Salmonella* genus, sequences conserved within the *Salmonella* genus, and those conserved across Enterobacteria (Table S1).

### TIS mutagenesis and HiBiT tagging

Endogenous 3’ HiBiT-tagged strains were constructed using λ red-mediated recombineering essentially following the protocol outlined by Datsenko and Wanner (77). Briefly, pORTMAGE-2-transformed *S*. *Typhimurium* cells, with λ red expression induced by incubating the culture for 15 min at 42°C in a warm water bath at 250 rpm, were made electrocompetent. Electroporation of these cells, grown to an OD_600_ of 0.6, was conducted with a linear PCR-editing substrate designed for HiBiT tagging and introducing the kanamycin- (*Km^R^*) or chloramphenicol- (*Cm^R^*) resistance cassette from pKD4 (accession number #7632, CGSS, Yale, USA) or pKD3, respectively (#45604, Addgene). PCR primers were designed to amplify the antibiotic-resistance cassette with 5’ and 3’ 50 bp homology arms complementary to the stop-codon flanking regions of *yebG*, *clpB*, *and fruK* using primers oPVDL2113 and oPVDL2114, oPVDL2191 and oPVDL2192, and oPVDL2309 and oPVDL2310, respectively (Table S3), and with the reverse primer incorporating an additional in-frame HiBiT tag (44) (11 aa tag MVSGWRLFKKIS). For TIS mutagenesis, a modified ssDNA oligo (40 pmol) was added to the electroporation reaction for multiplexed recombineering (*yebG*: oPVDL2063; *clpB*: oPVDL2496; *fruK*: oPVDL2419; Table S3). The ssDNA oligo contained two phosphorothioate linkages (indicated by an asterisk) (and a 2-fluoro-uridine modified base [2FU]) to create *yebG* aTIS (G-51A; Chr: 1,933,051 G > A), *fruK* dbTIS (GTG-1CTT; Chr: 2,303,857 – Chr: 2,303,859 GTG > CTT), and *clpB* aTIS (GTG-445CTT; Chr: 2,804,656 – Chr: 2,804,658 GTG > CTT) mutant strains exclusively expressing YebG^L^, FruK^L^, and ClpB^L^, respectively. Immediately following electroporation, bacterial cells were recovered in pre-warmed (28°C) Super Optimal broth with Catabolite repression (SOC, consisting of 2% tryptone, 0.5% yeast extract, 10 mM NaCl, 2.5 mM KCl, 5 mM MgCl_2_, 10 mM MgSO_4_, and 20 mM glucose) media at 28°C and were incubated at 180 rpm for 3 h. Mutant colonies were selected after plating the cell suspension and ~20 h of incubation (upside down) on LB agar plates supplemented with 50 µg/mL kanamycin or 25 µg/mL chloramphenicol at 28°C, and another round of re-streaking. The success of TIS mutagenesis and HiBiT tagging was confirmed through colony PCR using primer sequences indicated in Table S3 and through subsequent Sanger sequencing of the resulting PCR products (Eurofins Genomics).

### Total protein extraction and blotting analysis

WT and (TIS-mutated) HiBiT-tagged *S. Typhimurium* SL1344 strains (*yebG::HiBiT Km^R^*, *clpB::HiBiT Km^R^*, and *fruK::HiBiT Km^R^*) were cultured overnight in liquid cultures (OD_600_ 4–5). Subsequently, cultures were diluted 1:200 to an OD_600_ of 0.02 in 12-mL LB-Kan medium in a T25 cell culture flask with a ventilated cap. The flasks were incubated at 37°C with agitation (180 rpm) until reaching ESP (OD_600_ 2.0). Cultures (5 mL) (corresponding to ~1.25 10^9 bacterial cells) were harvested by centrifugation for 10 min at 6,000 *g* (4°C), and the supernatant was discarded. The bacterial pellets were washed in ice-cold D-PBS (1X Gibco, Cat. #14190144), transferred to a 1.5-mL Eppendorf tube, and centrifuged for 5 min at 6,000 *g*. Finally, the supernatant was discarded, and the bacterial pellets were stored at −80°C until further processing. The pellets were resuspended in 400 µL urea lysis buffer (9M urea, 50 mM ammonium bicarbonate NH_4_HCO_3_ [pH 7.9]) to achieve a resulting protein concentration of approximately 2 mg/mL. Next, samples were subjected to mechanical disruption by three cycles of freezing in liquid nitrogen and thawing in a water bath at room temperature. Additionally, two cycles of probe sonication (50% duty cycle; 30 s of sonication applying 1-s pulses) were executed using a Branson Sonifier 250 (Ultrasonic convertor) at an amplitude of 50. Cleared supernatant was obtained by centrifugation for 10 min at 16,000 *g* (4°C). Protein concentration was determined using the Bradford method (Bio-Rad DC Protein assay kit, Bio-Rad, Cat. #5000006) following the manufacturer’s instructions. 2X Tricine Sample Buffer (Bio-Rad, #1610739) containing 2% of β-mercaptoethanol was added to the samples, and equivalent amounts of total protein were analyzed by 1D SDS-PAGE on a 16.5% precast polyacrylamide Tris-Tricine-Criterion gel using 1X Tris/Tricine/SD buffer (100 mM Tris, 100 mM Tricine, 0.1% SDS, pH 8.3, 10X stock solution) (Bio-Rad, #1610744) at 150 V for 1 h 45 min. Proteins were subsequently transferred to a 0.45-µm nitrocellulose membrane for 30 min at 100 V using transfer buffer [380.71 mM Tris-HCl (Tris(hydroxymethyl)aminomethane hydrochloride) and 485.2 mM boric acid]. Next, the membrane was rinsed in Tris-buffered saline Tween-20 (TBS-T) (1X Tris-buffered saline [38.07 mM Tris-HCl and 148.87 mM NaCl] and 0.1% Tween-20, pH 7.5) for 15 min on an orbital shaking platform. LgBiT protein (Promega, Cat. #N401C; 1:200) was incubated with the membrane in TBS-T for 1 h at room temperature. Nano-Glo Luciferase Assay Substrate (Promega, Cat. #N113A; 1:2,000) was added and incubated for 5 min. Chemiluminescence was detected using the Odyssey LI-COR Fc infrared imaging system (LI-COR Biosciences, Odyssey Fc Imager model n° 2800). For subsequent loading control detection, the membrane was blocked for 30 min at room temperature using a 1:1 Intercept blocking buffer (LI-COR, Cat. #27-60001)/1X TBS-T, followed by 30-min incubation with streptavidin-Alexa Fluor 680 Conjugate (Invitrogen, Cat. #S32358; 1/5,000). Fluorescent detection of endogenously biotinylated protein served as the loading control and was performed using the Odyssey LI-COR Fc. Signal quantification was carried out using the Odyssey LI-COR Fc infrared imaging system analysis application (Empiria Studio Software), with normalization of the signal automatically conducted using a user-defined background area.

## Data Availability

The proteomics data used in this study have been deposited in the PRIDE repository under the accession number PXD029391. The Ribo-seq and Ribo-RET data sets and their associated metadata (16) are available through the Open Science Framework at https://osf.io/h3vxz/?view_only=8e69e3e04f2d43119595237436b42389, with a DOI of 10.17605/OSF.IO/H3VXZ, and at https://osf.io/3u2hd/?view_only=8fd6750f2eb44d9594aa89a69501ef9c, with a DOI of 10.17605/OSF.IO/3U2HD.

## SUPPLEMENTAL MATERIAL

Figure S1 mbio.00333-24-s0001.pdf Ribo-seq reveals translation of Nt-proteoform pairs in *S*. Typhimurium.

Figure S2 mbio.00333-24-s0002.tif Peptide detectability scores for the longest proteoform of identified N-terminal proteoform pairs.

Legends mbio.00333-24-s0003.docx Supplemental figure and table legends.

Table S2 mbio.00333-24-s0004.xlsx Physiochemical properties analyses of the members of identified N-terminal proteoform pairs identified in *S*. Typhimurium.

Table S3 mbio.00333-24-s0005.xlsx Primer sequences used.

ASM does not own the copyrights to Supplemental Material that may be linked to, or accessed through, an article. The authors have granted ASM a non-exclusive, world-wide license to publish the Supplemental Material files. Please contact the corresponding author directly for reuse.
