# Supplementary figures and images for: Proteins à la carte: riboproteogenomic exploration of bacterial N-terminal proteoform expression
Source: mBio. 2024 Mar 21;15(4):e00333-24. doi: 10.1128/mbio.00333-24 (PMC11005335; doi:10.1128/mbio.00333-24)

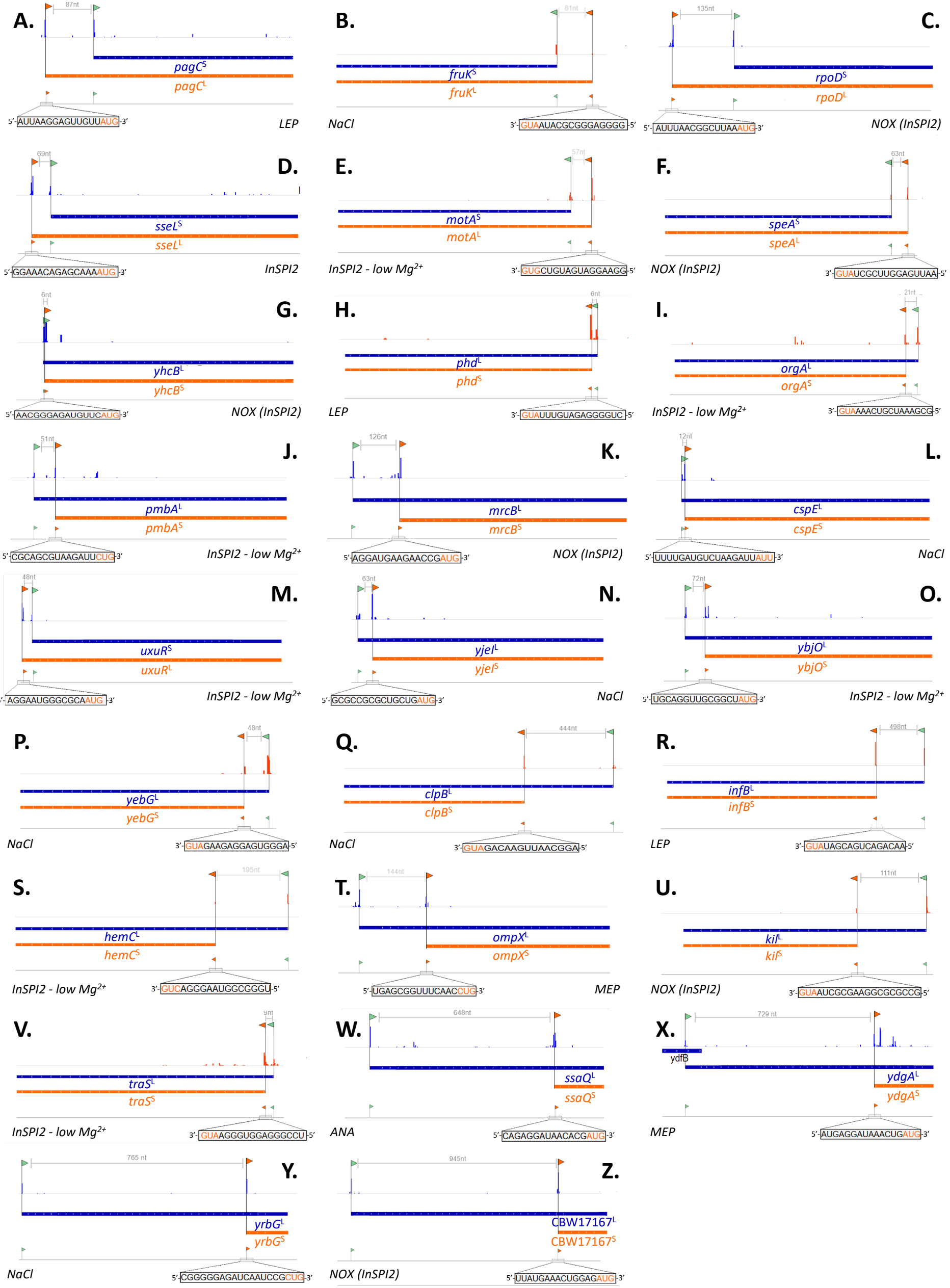

**Figure S1**

Supplement: Figure S1 — Ribo-seq reveals translation of Nt-proteoform pairs in S. Typhimurium. [file mbio.00333-24-s0001.pdf]

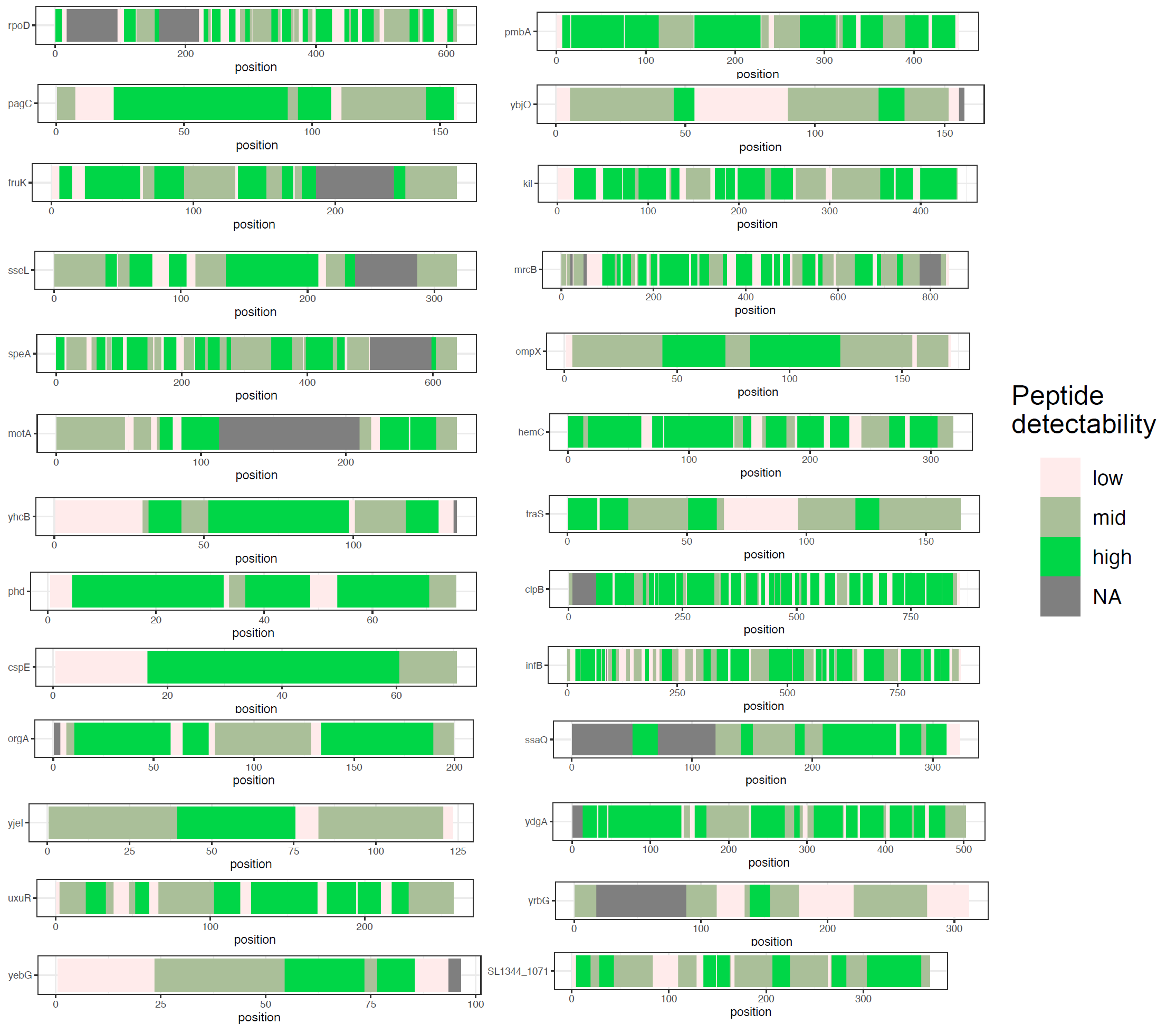

Supplement: Figure S2 — Peptide detectability scores for the longest proteoform of identified N-terminal proteoform pairs. [file mbio.00333-24-s0002.tif]
