# Supplementary material for: Proteins à la carte: riboproteogenomic exploration of bacterial N-terminal proteoform expression
Source: mBio. 2024 Mar 21;15(4):e00333-24. doi: 10.1128/mbio.00333-24 (PMC11005335; doi:10.1128/mbio.00333-24)
Supplement: Legends — Supplemental figure and table legends. [file mbio.00333-24-s0003.docx]

**Proteins à la carte: riboproteogenomic exploration of bacterial N-terminal proteoform expression.**

**APPENDIXES - Supplementary Figure and Table Legends**

**Supplementary Figure S1|** **Ribo-seq reveals translation of Nt-proteoform pairs in *S.* Typhimurium**. The Ribo-RET (RET) signal, indicative of (alternative) translation initiation, is displayed for all 26 confidently identified proteoform pairs (**Supplementary Table S1**). Called TIS are flagged, and the translation initiation region of alternative TIS (orange) is indicated. Representative data of different growth conditions, including mid-exponential growth phase (MEP), late exponential growth phase (LEP), salt stress condition (NaCl), anaerobic shock (ANA), nitric oxide shock in SPI2-inducing PCN (NOX (InSPI2)), SPI2-inducing PCN (InSPI2), and low magnesium SPI2-inducing PCN (low Mg^2+^), are presented.

**Supplementary Figure S2| Peptide detectability scores for the longest proteoform of identified N-terminal proteoform pairs.** AP3-derived detectability scoring (1) is represented in a color-code according to the AP3 scale: high detectability (scores greater than 0.9) is indicated in green, medium detectability (scores between 0.4 and 0.9) in light green, and low detectability (scores less than 0.4) in pale red.

**Supplementary Table S1| Ribo-seq investigation of expressed N-terminal proteoform pairs in *S.* Typhimurium.** All 26 confident expressed alternative proteoforms from identified proteoform pairs are listed together with their corresponding gene names, chromosomal location, strand (+ or -), corresponding translation initiation start (TIS) codon, ORF length (in nucleotides (nt.), proteoform type (N-terminally extended (ext) of N-terminally truncated (trunc) proteoform), distance of alternative TIS (aTIS) to database annotated TIS (dbTIS), Predicted PSORTb localization (OMP – outer membrane protein, IMP – inner membrane protein, PP – periplasmic protein), binding energies of aTIS and dbTIS, quantitative Ribo-RET translation dbTIS and aTIS measurements across the seven conditions analyzed, and identified proteogenomic peptides. The conditions represented include mid-exponential growth phase (MEP), late exponential growth phase (LEP), salt stress condition (NaCl), anaerobic shock (ANA), nitric oxide shock in SPI2-inducing PCN (NOX (InSPI2)), SPI2-inducing PCN (InSPI2), and low magnesium SPI2-inducing PCN (low Mg^2+^).

**Supplementary Table S2| Physiochemical properties analyses of identified N-terminal proteoform pairs in *S.* Typhimurium.** All 26 confident expressed proteoform pairs are listed together with their corresponding gene names, amino acid (aa) sequences and lengths, TIS category (alternative TIS (aTIS) or database annotated TIS (dbTIS)), proteoform type (N-terminally extended (long) of N-terminally truncated (short) proteoform), calculated physiochemical properties (GRAVY, isoelectric point (pI), Instability index, Aliphatic index, molecular weight (MW)), and the distance between alternative (aTIS) and database annotated (dbTIS) in amino acids (aa).

**Supplementary Table S3| Primer sequences used.**

**References**

1. Gao Z, Chang C, Yang J, Zhu Y, Fu Y. 2019. AP3: An Advanced Proteotypic Peptide Predictor for Targeted Proteomics by Incorporating Peptide Digestibility. Anal Chem 91:8705-8711.
